# Histologic Heterogeneity of Extirpated Renal Cell Carcinoma Specimens: Implications for Renal Mass Biopsy

**DOI:** 10.15586/jkcvhl.2020.134

**Published:** 2020-08-25

**Authors:** Lauren M. Nahouraii, Jordan L. Allen, Suzanne B. Merrill, Erik Lehman, Matthew G. Kaag, Jay D. Raman

**Affiliations:** 1 College of Medicine, Pennsylvania State University, Hershey, PA, USA;; 2 Division of Urology, Department of Surgery, Penn State College of Medicine, Hershey, PA, USA;; 3 Department of Public Health Sciences, Penn State College of Medicine, Hershey, PA, USA

**Keywords:** heterogeneity, histology, renal cell carcinoma, renal mass biopsy, renal tumors

## Abstract

Pathologic characteristics of extirpated renal cell carcinoma (RCC) specimens <7 cm were reviewed to get better information on technical nuances of renal mass biopsy (RMB). Specimens were stratified according to tumor stage, nuclear grade, size, histology, presence of lymphovascular invasion (LVI), necrosis, and sarcomatoid features. When considering pT1 (0–7 cm) tumors, pT1b (4–7 cm) RCC masses were more likely to have necrosis (43% vs 16%, P < 0.001), LVI (6% vs 2%, P = 0.024), high-grade nuclear elements (29% vs 17%, P < 0.001), and sarcomatoid features (2% vs 0%, P = 0.006) compared with pT1a (0–4 cm) tumors. Additionally, pT3a tumors were more highly associated with necrosis (P = 0.005), LVI, sarcomatoid features, and high-grade disease (P for all < 0.001) when compared to pT1 masses. For masses <4 cm, pT3a cancers were more likely to demonstrate necrosis (38% vs 16%, P < 0.001), LVI (22% vs 2%, P < 0.001), high-grade nuclear elements (45% vs 17%, P < 0.001), and sarcomatoid features (12% vs 0%, P < 0.001) compared to pT1a tumors. Similarly, for masses 4–7 cm, pathologic T3a tumors were significantly more likely to have sarcomatoid features (12% vs 2%, P = 0.006) and LVI (22% vs 6%, P = 0.003) compared to pT1b tumors. In summary, pT3a tumors and those RCC masses >4 cm exhibit considerable histologic heterogeneity and may harbor elements that are not easily appreciated with limited renal sampling. Therefore, if RMB is considered for renal masses greater than 4 cm or those that abut sinus fat, a multi-quadrant biopsy approach is necessary to ensure adequate sampling and characterization of the mass.

## Introduction

Concerns regarding insufficient diagnostic yield coupled with false positive and negative rates contributed to the initial low utilization of renal mass biopsy (RMB) as a diagnostic tool for cortical masses over a decade ago. However, the rise of small renal masses discovered incidentally on imaging and advancements in systemic agents against particular renal cell carcinoma (RCC) subtypes have led to a resurgence of interest in incorporating RMB into the diagnostic algorithm of renal tumors (1). Furthermore, there has been growing interest in the use of genetic and molecular assessments that can potentially improve our ability to determine renal tumor biology and clinical behavior through RMB (2). Additionally, the increased adoption for active surveillance across a spectrum of RCC masses renewed optimism in the value of RMB information (3).

Current American Urological Association (AUA) clinical principles state that RMB should be considered when a mass is suspected to be hematologic, metastatic, inflammatory, or infectious. In the setting of a solid renal mass, AUA expert opinion does not recommend RMB for young or healthy patients who are willing to accept uncertainties associated with RMB, or for older or frail patients who will be managed conservatively regardless (4). Conversely, the European Urological Association practice guidelines recommend the use of RMB in patients who are candidates for active surveillance of small masses, to obtain histology before ablative treatments, and to select the most suitable medical and surgical treatment strategy in the setting of metastatic disease (level 3 evidence) (5).

The ideal RMB would not only differentiate between benign and malignant tumors, but importantly characterize key histologic features of RCC tumors to better guide the use of conservative versus interventional strategies. RMB is overall a safe procedure with core biopsy having superior accuracy compared to final needle aspiration (FNA) (6). There is some variability, however, in diagnostic accuracy particularly with respect to the impact of intratumoral heterogeneity on the interpretation of pathologic outcomes (6). Therefore, we evaluated the incidence of histologic heterogeneity, necrosis, lymphovascular invasion (LVI), and sarcomatoid features, in extirpated RCC specimens to better guide specific nuances of the RMB technique to optimize interpretation and accuracy.

## Patients and Methods

After institutional review board (IRB) approval, the records of patients who underwent surgical resection of renal masses at a single academic institution between January 2000 and August 2017 were reviewed. Patients having incomplete data and benign masses were excluded. Masses >7 cm were excluded due to the likelihood of extirpation versus diagnostic biopsy in general clinical practice. The baseline demographic, disease, and treatment-related parameters were extracted from the records in a de-identified manner. These data included age, gender, race, body mass index (BMI), American Society of Anesthesiologists (ASA) score, Eastern Cooperative Oncology Group (ECOG) performance status, and type of surgery (radical versus partial nephrectomy).

Final pathology reports of RCC specimens were used to stratify the masses according to size (0–4 cm and 4–7 cm) and pathologic stage (pT1a, pT1b, and pT3a). Within these groups, the annotation was made regarding histologic elements including nuclear grade and presence of LVI, necrosis, and sarcomatoid features (7). Low- and high-grade nuclear elements were defined as Fuhrman grades 1–2 and 3–4, respectively.

The data were analyzed using R software, version 9. The categorical variables were compared using Chi square test with Bonferroni correction to determine significance at a level of P < 0.05.

## Results

A total of 659 RCC specimens met the inclusion criteria. The pathologic characteristics of our cohort are included in Table 1. In summary, the mean tumor size was 3.5 cm (range, 0.9–7.0 cm). Pathologic distribution of tumors included 404 (61%) pT1a, 169 (26%) pT1b, and 86 (13%) pT3a. Histologic subtypes were distributed as 74% clear cell, 19% papillary, 6% chromophobe, and 1% other. Specimen stratification revealed 66% of tumors 0–4 cm and 34% of tumors 4–7 cm with no statistical difference in histologic subtype distribution across renal tumor sizes. Low and high nuclear grades were recorded in 486 (74%) and 156 (24%) cases, respectively. Necrosis, LVI, and sarcomatoid features occurred in 26, 6, and 2% of cases, respectively.

**Table 1:** Pathologic characteristics of 659 RCC tumors ≤ 7cm in size

Pathologic Variable	No. (%)
Histologic Subtype Clear Cell Papillary Chromophobe	487 (74) 128 (19) 38 (6)
Tumor Size (cm) 0 – 4 4 – 7	433 (66) 226 (34)
Tumor Stage pT1a pT1b pT3a	404 (61) 169 (26) 86 (13)
Nuclear Grade Low High	486 (76) 156 (24)
Necrosis	171 (26)
Lymphovascular Invasion	38 (6)
Sarcomatoid features	14 (2)

When considering pathologic T1 tumors, pT1b lesions (4–7 cm in size) were more likely to have necrosis (43% vs 16%, P < 0.001), LVI (6% vs 2%, P = 0.024), high-grade nuclear elements (29% vs 17%, P < 0.001), and sarcomatoid features (2% vs 0%, P = 0.006) when compared to pT1a tumors (0–4 cm in size) (Table 2).

**Table 2:** Pathologic characteristics of 573 pT1 tumors stratified by size (0–4cm vs. 4–7cm)

Pathologic Variable	pT1a (n=404) No. (%)	PT1b (n=169) No. (%)	P value
Histologic Subtype Clear Cell Papillary Chromophobe	297 (74) 81 (19) 26 (6)	127 (75) 33 (20) 6 (4)	1.0 1.0 1.0
Nuclear Grade Low High	326 (81) 68 (17)	114 (67) 49 (29)	<0.001
Necrosis	66 (16)	72 (43)	<0.001
LVI	9 (2)	10 (6)	0.024
Sarcomatoid features	0 (0)	4 (2)	0.006

Pathologic pT3a tumors were more highly associated with necrosis (P=0.005), LVI, sarcomatoid features, and high-grade disease (P for all <0.001) when compared to pT1 masses (Table 3). Specifically, in masses <4 cm, pT3a cancers were more likely to demonstrate necrosis (38% vs 16%, P < 0.001), LVI (22% vs 2%, P < 0.001), high-grade nuclear elements (45% vs 17%, P < 0.001), and sarcomatoid features (12% vs 0%, P < 0.001) compared to pT1a tumors. For masses 4–7cm, pathologic pT3a tumors were significantly more likely to have sarcomatoid features (12% vs 2%, P < 0.006) and LVI (22% vs 6%, P < 0.001) compared to pT1b tumors (Table 4).

**Table 3:** Pathologic characteristics of 659 RCC tumors < 7cm in size stratified by stage (pT1 vs. pT3a)

Pathologic Variable	pT1 (n=573) No. (%)	PT3a (n=86) No. (%)	P value
Histologic Subtype Clear Cell Papillary Chromophobe	424 (74) 114 (20) 32 (6)	63 (73) 14 (16) 6 (7)	1.0 1.0 1.0
Nuclear Grade Low High	440 (77) 117 (20)	46 (53) 39 (45)	<0.001
Necrosis	138 (24)	33 (38)	0.005
LVI	19 (3)	19 (22)	<0.001
Sarcomatoid features	4 (1)	10 (12)	<0.001

**Table 4:** Pathologic characteristics of 659 RCC tumors ≤ 7cm in size stratified by stage (pT1 vs. pT1b vs. pT3a)

Pathologic Variable	pT1a (n=573) No. (%)	pT1b (n=169) No. (%)	PT3a (n=86) No. (%)	P value
Histologic Subtype Clear Cell Papillary Chromophobe	297 (74) 81 (19) 26 (6)	127 (75) 33 (20) 6 (4)	63 (73) 14 (16) 6 (7)	0.94 0.72 0.35
Nuclear Grade Low High	326 (81) 68 (17)	114 (67) 49 (29)	46 (53) 39 (45)	<0.001
Necrosis	66 (16)	72 (43)	33 (38)	<0.001
LVI	9 (2)	10 (6)	19 (22)	<0.001
Sarcomatoid features	0 (0)	4 (2)	10 (12)	<0.001

## Discussion

Renal mass biopsy is a tool increasingly available for accurate characterization of renal pathology prior to definitive intervention. While histologic heterogeneity of RCC is recognized, a quantitative assessment is more limited. Here, we investigated the pathologic characteristics of 659 extirpated RCC specimens to better characterize various elements that may impact the disease biology. In this regard, we observed RCC masses >4 cm and pT3a lesions, irrespective of size, were more likely to harbor areas of necrosis, LVI, high-grade nuclear elements, and sarcomatoid features.

Several other investigators have similarly investigated histologic characteristics of kidney cancers. Bedke et al. (8) demonstrated LVI in 5.5% of subjects with RCC and showed an association with advanced TNM (tumor, lymph node, metastasis) stages, high Fuhrman grade, and sarcomatoid features. LVI was the strongest factor associated with metastatic spread in all patients and also in a subgroup of patients with clear cell RCC. Pichler et al. (9) reported that sarcomatoid features, a highly aggressive form of RCC, were present in 5–8% of clear-cell RCC, 8–9% of chromophobe RCC, and 2–3% of papillary RCC. Additionally, about 75% of patients with sarcomatoid RCC presented with metastatic disease. Khor and colleagues (10) noted that tumor necrosis was present in 21% of cases of resected renal masses. After adjusting for age, tumor stage, and tumor size, necrosis was a significant predictor of overall survival on the multivariable analysis.

Nuclear grade heterogeneity is known to occur in small renal masses. In particular, Ball and colleagues (7) observed Fuhrman grade heterogeneity in 81.3% of all RCC tumors and 93.8% of high-grade specimens. Additionally, they investigated discordance within RCC specimens, defined as the highest grade is present in less than 50% of the specimen. They found discordance among 31.3% of all RCC tumors and 25% of high-grade specimens. A small-scale study confirmed that there is a statistically significant difference in the proportion of high-grade cases when compared with conventional (36%) and total (60%) samplings (11).

Qualitative data comparing histologic heterogeneity based on size and stage were limited in our literature review. Zhang et al. (12) showed a significant correlation between tumor size with grade and stage of lesions. Specifically, larger tumors were prone to have a higher grade and stage, and the probability of being clear cell subtype grew higher as the tumor size increased. In a recent study of a multi-quadrant biopsy technique for large renal masses (T2 or greater), 25.0% revealed multiple grades of tumors present among the cores, including 14.1% that contained high- and low-grade tumors. Of the 13 patients with sarcomatoid features identified from multi-quadrant biopsies, only 30.8% had sarcomatoid features present in every core sampled (13).

Heterogeneity is well established in RCC at the chromosomal and genomic sequence level. Moch et al. demonstrated variable intratumor loss of Von Hippel-Lindau (VHL) and chromosome 3 polysomy by fluorescence in situ hybridization in 53 patients with pT2 or greater RCC. However, the cytogenetic variability seen in these cases did not correlate with grade or stage (2, 14). Likewise, using multiregional exome sequencing, Gerlinger et al. (15) demonstrated intratumor heterogeneity in somatic mutations and genomic copy number across multiple regions in primary tumors and metastatic lesions in patients with clear cell RCC. Further research continues to identify genetic mutations with a recent meta-analysis tallying more than 19 (16).

Given the intratumoral heterogeneity demonstrated in this study, the probability of identifying all pathological features increases with each area tested by reducing sampling error. Hobbs et al. (17) demonstrated in an ex vivo study that obtaining multiple renal mass biopsy cores, compared with a single core biopsy, considerably improved diagnostic yield for histologic subtyping, accuracy, and cancer identification. Second peripheral and second central cores both increased cancer identification versus a single peripheral core (56– 77% and 80%, respectively). They found no difference in cancer identification based on the location of a single core (central vs peripheral). These findings were reinforced by Abel et al. (13) in a prospective, in vivo study of multi-quadrant biopsies of large renal masses (T2 or greater). The use of at least four versus three or fewer biopsies from separate, solid enhancing areas of tumor decreased nondiagnostic rate (11 to 0%) and improved sensitivity for detecting sarcomatoid features (25 to 87%) without increasing the rate of complications. Multicenter studies are needed to confirm these findings.

There are limitations to this study. It is retrospective in nature and examines prior pathology reports. As such, we only have reports of the highest Fuhrman grade of an entire extirpated mass. Additionally, our institution currently follows the International Society of Urological Pathology 2018 guidelines requiring one section for each centimeter of the largest diameter of the tumor, with special attention to the areas with different macroscopic aspects including necrosis, but no precise guidelines for the distribution of sampling. The problem is that many RCC that appear homogeneous to the naked eye are heterogeneous under the microscope. Necrosis and sarcomatoid transformation can be discovered sometimes on a meticulous and skilled analysis by the naked eye, but tumor grade and eosinophilic cell change cannot (18). This point makes current routine tumor sampling an inconsistent sampling method (19, 20).

Furthermore, there are no standard recommendations on how renal carcinomas should be graded in needle-core biopsies. At our institution, we assign the highest grade to total specimen, but a system similar to Gleason scoring could prove more useful for stage stratification and treatment decisions. Further research is necessary to incorporate RMB into a standard diagnostic protocol for patients with renal cortical tumors.

## Conclusion

Renal cell carcinoma masses >4 cm and pT3a tumors (irrespective of size) exhibit considerable histologic heterogeneity and may harbor elements that are not easily appreciated with limited renal sampling. Therefore, if by cross-sectional radiography, renal masses appearing larger than 4 cm or those abutting sinus fat, a single percutaneous biopsy sample is likely not adequate. A multi-quadrant biopsy is necessary for these tumors to ensure accurate capture of histologic elements that may better reflect the differing underlying biology of these tumors. The minimum number and specific location of biopsies require continued investigation.
